# Phosphodiesterase 3 A expression in gastrointestinal stromal tumors

**DOI:** 10.1007/s00428-025-04150-1

**Published:** 2025-06-18

**Authors:** Harri Sihto, Olivier Giger, Kirsi Toivanen, Sami Salmikangas, Tiina Vesterinen, Mika Sampo, Tom Böhling

**Affiliations:** 1 Rare Cancers Research Group, Department of Pathology, University of Helsinki and Helsinki University Hospital, Haartmaninkatu 3, Helsinki, FI-00014 Finland; 2 Department of Pathology, University of Cambridge, Addenbrooke’s Hospital, Cambridge, UK; 3 HUS Diagnostic Center, Department of Pathology, University of Helsinki and Helsinki University Hospital, Helsinki, Finland

**Keywords:** Gastrointestinal stromal tumor, Phosphodiesterase 3A, Immunohistochemistry, Monoclonal antibody

## Abstract

Phosphodiesterase 3A (PDE3A) is an emerging therapy target in various cancers with high expression, as in the majority of gastrointestinal stromal tumors (GISTs). However, its association with clinicopathological factors and patient survival in GISTs remains unexplored. We investigated PDE3A expression using a novel mouse monoclonal antibody and immunohistochemistry in two GIST patient series consisting of 173 formalin-fixed, paraffin-embedded tissue samples on tissue microarrays. In addition, we analyzed the association between PDE3A staining intensity and clinicopathological variables and patient survival. We also assessed PDE3A mRNA expression using qPCR in a subset of the samples. We found that all GISTs expressed PDE3A. The staining pattern was weak in 6.3%, intermediate in 35.8%, and strong in 57.8% of tumors. Weak PDE3A expression was associated with a lower median mitotic count (1/50 HPF vs. 4/50 HPF vs. 5/50 HPF; *p* = 0.007) and higher incidence of metastases at diagnosis (28% vs. 8% vs. 3.3%; *p* = 0.040) than in tumors with intermediate or strong expression, respectively. PDE3A and CD117 staining intensities correlated positively (*p* < 0.001), but PDE3A expression showed no association with sex, age, tumor size, location, risk stratification, mutation profile, Schlafen 12 expression, or metastasis-free or overall survival. We conclude that PDE3A expression can be reliably assessed in archived tumor material using immunohistochemistry. GISTs, with their consistently high PDE3A expression, are a promising target for PDE3A-targeted therapies. This method may also aid in stratifying patients in cancers where PDE3A expression is less common.

**Supplementary Information:**

The online version contains supplementary material available at 10.1007/s00428-025-04150-1.

## Introduction

Gastrointestinal stromal tumor (GIST) is the most common gastrointestinal soft-tissue sarcoma with an annual incidence of 10 to 20 persons per million persons [1, 2]. GIST is characterized by mutually exclusive gain-of-function mutations in *KIT* and *platelet-derived growth factor receptor alpha* (*PDGFRA)* oncogenes in ~ 85% of cases [1, 3]. These mutations in receptor tyrosine kinase-encoding genes lead to ligand-independent constitutive activation of the kinases, driving uncontrolled tumor cell proliferation and survival [4, 5]. In addition, the mutated receptors serve as key therapeutic targets for tyrosine kinase inhibitors such as imatinib, sunitinib, regorafenib, ripretinib, and avapritinib [6]. However, the specific mutation type has a marked impact on therapy sensitivity of GISTs, and secondary resistance to therapies frequently evolves during treatment [7].

Approximately 10–15% of GISTs do not harbor *KIT* or *PDGFRA* mutations and are generally insensitive to tyrosine kinase inhibitors. The most common tumorigenic aberrations in these GISTs include deleterious mutations in succinate dehydrogenase (SDH) enzyme complex subunit genes *SDHA*, *SDHB*, *SDHC,* and *SDHD* and gene silencing epimutations of *SDHC* [8]*.* Additionally, rare pathway-activating driver mutations in the RAS/RAF/MAPK pathway, affecting genes such as *BRAF* and *NF1*, and *ETV6-NTRK3* fusions have been reported [1, 9]. Importantly, in these rare cases, *BRAF* mutations and *NTRK* fusions may enable targeted therapy with specific inhibitors, such as NTRK inhibitors larotrectinib and entrectinib, and BRAF or combined BRAF and MEK targeting therapies [6].

Phosphodiesterase 3 A (PDE3A) is an emerging therapeutic target in GIST. Previous studies indicate that 90–100% of GISTs express PDE3A, and its expression is high compared with other tumor types or healthy tissues [10–12]. PDE3A is also expressed in interstitial cells of Cajal (ICC), which are the presumed precursors of GIST and play a role in regulating ICC development and network density in the gastrointestinal tract [13]. Interestingly, PDE3A silencing or inhibition of its enzyme activity does not impair the viability of GIST cells [11]. Instead, PDE3A has a structural role as a protein complex partner with Schlafen 12 (SLFN12), which is co-expressed in 92% of GISTs [13]. PDE3A modulators, such as anagrelide, nauclefin, and DNMDP, act as molecular glues, inducing protein–protein interaction between PDE3A and SLFN12. The formation of the PDE3A-SLFN12 complex leads to increased cytoplasmic SLFN12 stability and ultimately cell death-inducing RNase activity in the cells [14–16]. Notably, two of these modulators, anagrelide and OPB-171775, have demonstrated significant anti-tumor efficacy in patient-derived GIST xenograft mouse models, including those resistant to tyrosine kinase inhibitors [11, 17]. Additionally, both compounds have shown synergy with imatinib in GIST PDX mouse models [11, 17].

Although several studies have reported high PDE3A expression in GISTs, its potential role as a prognostic or diagnostic biomarker remains uncharacterized. In this study, we examined PDE3A expression in 173 formalin-fixed paraffin-embedded (FFPE) GIST tumor samples using a novel mouse monoclonal antibody and immunohistochemistry (IHC). We also investigated the correlation between PDE3A mRNA and protein expression. Additionally, we analyzed the association between PDE3A expression, GIST genotype, clinicopathological factors, SLFN12 expression and survival.

## Material and methods

### Sample series

Two retrospective series of FFPE GIST tissue samples were analyzed. The first series, collected from the Helsinki Biobank (Helsinki, Finland), included 161 samples. Tumors were diagnosed at Helsinki University Hospital (HUH) between 1990 and 2020, and corresponding clinical data (Table 1) were retrieved from the HUH data lake, as described earlier [18]. Metastasis-free survival data were updated from radiology reports, with 53 cases missing this information (data collection closed on 18 April 2023). Fourteen cases were excluded due to missing clinical information, misdiagnosis after histological re-evaluation, insufficient tissue, or samples not from primary tumors, leaving 147 cases for analysis. Tissue microarrays (TMAs) were constructed from three to six 1.0 mm core punches per tumor sample. Mutations in *KIT* exons 9, 11, 13, and 17 and *PDGFRA* exons 12 and 18 were analyzed by Sanger sequencing, and CD117 expression was assessed via immunohistochemistry on TMA, as described elsewhere [18]. GISTs were stratified into risk categories based on the Modified National Institutes of Health (M-NIH) system, omitting tumor rupture due to missing data [19]. Nine patients had metastases at diagnosis.

**Table 1:** PDE3A expression and tumor characteristics in 147 GISTs

Characteristic	PDE3A expression			*P*-value
	Low (*n* = 7)	Intermediate (*n* = 50)	High (*n* = 90)	
Sex, *n* (%)				0.584
Male (*n* = 73)	3 (42.9)	28 (56.0)	42 (46.7)	
Female (*n* = 74)	4 (57.1)	22 (44.0)	48 (53.3)	
Median age at diagnosis (range), years	57 (50–75)	67 (36–91)	65 (10–89)	0.434*
Median tumor diameter (range), cm	5.5 (2.5–8.5)	6.0 (0.9–22.0)	5.1 (1.0–29.0)	0.579*
NA		2	2	
Median mitotic count per 50 HPF (range)	1 (0–5)	4 (0–39)	5 (0–100)	0.007*
NA		1	2	
Location, *n* (%)				
Gastric (*n* = 105)	5 (71.4)	35 (70.0)	66 (73.3)	0.938
Intestinal (*n* = 35)	2 (28.6)	13 (26.0)	20 (22.2)	
Extragastrointestinal (n = 7)	0 (0)	2 (4.0)	4 (4.4)	
Risk category, *n* (%)				
Very low (*n* = 4)	0 (0)	3 (6.8)	1 (1.2)	0.500
Low (*n* = 45)	1 (25.0)	14 (31.8)	29 (34.9)	
Intermediate (*n* = 28)	2 (50.0)	9 (20.5)	17 (20.5)	
High (*n* = 62)	1 (25.0)	18 (40.9)	36 (43.4)	
NA	1	2	4	
Metastasis at diagnosis, *n* (%)				
Absent (*n* = 138)	5 (71.4)	46 (92.0)	87 (96.7)	**0.040**
Present (*n* = 9)	2 (28.6)	4 (8.0)	3 (3.3)	
Mutated gene and exon, *n* (%)	>			0.222
KIT/PDGFRA wild type (*n* = 22)	1 (14.3)	6 (12.0)	15 (16.7)	
KIT exon 9 (*n* = 11)	1 (14.3)	5 (10.0)	5 (5.6)	
KIT exon 11 (*n* = 85)	4 (57.1)	23 (46.0)	59 (65.6)	
KIT exon 13 (*n* = 6)	0 (0)	2 (4.0)	3 (3.3)	
PDGFRA exon 12 (*n* = 2)	0 (0)	2 (4.0)	0 (0)	
PDGFRA exon 18 (*n* = 17)	1 (14.3)	9 (18.0)	7 (7.8)	
NA		3	1	
CD117 staining intensity, *n* (%)				** < 0.001**
Negative	1 (14.3)	0 (0)	0 (0)	
Weak	1 (14.3)	9 (18.0)	3 (3.3)	
Intermediate	2 (28.6)	12 (24.0)	5 (5.6)	
Strong	3 (42.9)	29 (58.0)	82 (91.1)	
Schlafen 12 staining, *n* (%)				0.083
Negative	4 (57.1)	12 (24.0)	35 (38.9)	
Positive	3 (42.9)	38 (76.0)	55 (61.1)	

*NA* Not available

*Association investigated between continuous variables and PDE3A expression using the Kruskal–Wallis test

The second GIST series consists of 26 FFPE tumor samples on TMAs, collected at the National Paediatric and Adult Wild-Type GIST and GIST Clinic at Cambridge University Hospitals NHS Foundation Trust, UK. Their mutations were analyzed using Sanger sequencing, as described elsewhere [20]. The tumors harbored mutations in the following genes: *KIT* (*n* = 6), *PDGFRA* (*n* = 4), *NF1* (*n* = 4), or one of the *SDH* genes (*SDHA, SDHB, SDHC,* SDHD, n = 12).

The study on the GIST and liposarcoma series collected from Helsinki Biobank was approved by the Ethics Committee IV of HUH (HUS/1258/2020). Permission to use tissue samples and clinical data was granted by Helsinki Biobank (HUS/430/2021 §4) and HUH (HUS/244/2021). Project-specific consent for this retrospective study was waived since the Finnish Biobank Act provides a lawful basis for research use. The study of GIST samples collected from Cambridge was approved by Cambridge South Research Ethics Committee (REC reference number CA/5175) and all patients provided informed consent.

### Generation of the antibody

Mouse monoclonal anti-PDE3A antibodies were produced by immunizing mice with a peptide (amino acids 669–1141) from the catalytic domain of PDE3A (ProteoGenix SAS, Schiltigheim, France) and provided for the study by Sartar Therapeutics Ltd. (Helsinki, Finland). After spleen cell fusion and screening of hybridoma supernatants by ELISA, 99 positive clones were identified. From these, the supernatants of the 20 clones with the highest binding affinity were selected for testing in immunohistochemistry on FFPE colon tissue, using interstitial cells of Cajal as a positive control for specific staining. The four clones that showed the highest specificity were subcloned and monoclonal antibodies affinity-purified and further investigated using immunohistochemistry. Finally, clone 230-F8-G1 was selected to be used in immunohistochemistry.

### Immunohistochemistry

TMA Sects. (3 µm thick) were placed on Superfrost® Plus objective slides (Thermo Scientific, MA, USA) and warmed at 56 °C for 30 min following paraffin removal in xylene, followed by a graded alcohol-to-water wash. Endogenous peroxidase activity was blocked by incubating the slides in 0.8% hydrogen peroxide for 30 min. Heat-induced epitope retrieval was performed using EnVision FLEX Target Retrieval LOW Solution (Cat# K8005, pH 6.0; Dako, CA, USA) in a HIER Decloaking Chamber (Biocare Medical, CA, USA) at 95 °C for 15 min.

The PDE3A antibody was diluted to 9.2 µg/mL in ImmunoLogic Normal Antibody Diluent (Cat# BD09-500, WellMed, Duiven, Netherlands) and incubated on tissues for 30 min at room temperature in a humidity chamber. A secondary antibody (BrightVision 1-step detection system rabbit HRP, Cat# DPVM110HRP, WellMed) was then applied for 30 min under the same conditions. Antibody binding was detected using the ImmPACT DAB Substrate kit (Cat# SK4105, Vector Laboratories, CA, USA) for 5 min. SLFN12 expression was assessed as previously described, using a polyclonal SLN12 rabbit antibody (Cat# PA5-114360, Thermo Scientific) [21]. Finally, tissues were counterstained with Mayer’s Hematoxylin (Cat# S3309, Dako) for 3 min at room temperature, then dehydrated through a graded alcohol series to xylene. Sections were washed with Tris-buffered saline containing 0.1% Tween-20 before and after antibody incubation steps. CD117 expression was assessed as described elsewhere [18].

Syncytiotrophoblasts in placental tissue served as a positive control for SLFN12 staining, and staining intensity was categorized as either negative or positive. Interstitial cells of Cajal in colon tissue served as a positive control for PDE3A staining. Bone marrow tissue for megakaryocyte PDE3A expression was also assessed. The stained slides were digitalized using a 20 × objective and with a Panoramic scanner (3DHISTECH, Budapest, Hungary) in Helsinki Biobank and scored manually using SlideViewer software (version 2.7.0.191696, 3DHISTECH). Due to the homogeneous staining pattern, PDE3A staining intensity was graded into four categories: absent, low, intermediate, and strong (0 to 3). GISTs were analyzed independently by three pathologists (O.G., M.S., T.B.). For statistical analysis, the average of the scores was calculated and rounded to the nearest whole number.

### RT-qPCR

RNA was extracted from 30 GIST and 10 liposarcoma FFPE tissue sections using QIASymphony RNA kit and QIASymphony SP instrument (Qiagen GmbH, Hilden, Germany), and 2 µL of RNA (30 to 300 ng) was reverse-transcribed into cDNA using SuperScript™ VILO™ cDNA synthesis kit (Cat# 11754250, Thermo Ficher Scientific, Carlsbad, CA, USA) following the manufacturer’s instructions, as described earlier [12, 18]. RNA extracted from the FFPE sample made from the GIST882 cell line was used as a reference sample. Quantitative PCR was conducted using QuantiNova Probe PCR kit (Cat# 208254, Qiagen), and primers and probe in a ready-to-use mixture for *PDE3A* expression were designed with QuantiNova LNA PCR software (https://geneglobe.qiagen.com/fi/customize/pcr/mrna-incrna/quantinova-lna-probe-pcr-custom-assays). *YWHAZ* served as a reference gene (Forward: 5´-CGT TAC TTG GCT GAG GTT GC-3’; Reverse: 5’ -TGC TTG TTG TGA CTG ATC GAC-3’; Probe #9: Cat# 04683633001, Universal ProbeLibrary, Roche, Basel, Switzerland).

Real-time PCR was performed using the CFX96 Real-Time System (Bio-Rad Laboratories Inc., Hercules, CA, USA) to triplicate samples under following cycling: initial denaturation at 95 °C for 2 min, followed by 50 cycles at 95 °C for 30 s and at 60 °C for 30 s with plate reading. Relative PDE3A mRNA expression was calculated using the ΔΔCt method and normalized to YWHAZ expression.

### Statistical analyses

Crosstabs were analyzed using either Fisher’s exact test or Fisher–Freeman–Halton test. The association between PDE3A expression and continuous variables was analyzed using the Kruskal–Wallis test. Inter-rater concordance was estimated using Fleiss’ kappa analysis. Overall survival was calculated from the date of diagnosis to the date of death, and metastasis-free survival from the date of diagnosis to the date of detected metastasis, censoring the patients without events at the end of follow-up. Survival was estimated using the Kaplan–Meier method and compared using a log-rank test. Statistical analyses were performed using IBM SPSS Statistics version 29.

## Results

### PDE3A protein expression in GIST sample series

**Fig. 1:**
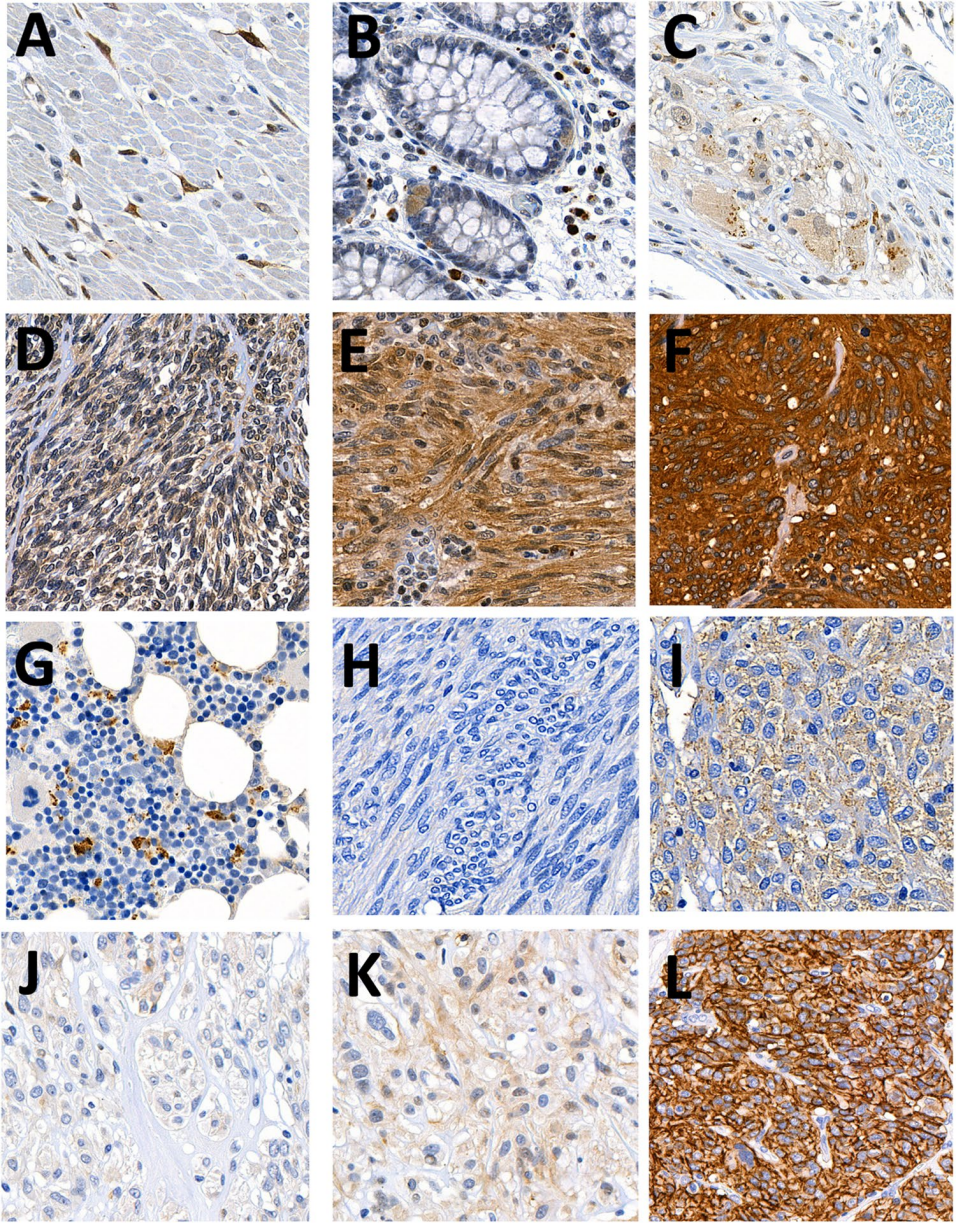
Examples of immunohistochemical stainings in healthy and tumor tissues. PDE3A expression in (**A**) Interstitial cells of Cajal, **B** neuroendocrine epithelial cells and mast cells, and (**C**) ganglia cells in colon. Examples of (**D**) weak, **E** intermediate, and **F**) strong staining of PDE3A in GISTs. **G** PDE3A staining was absent in megakaryocytes and showed granular staining in histiocytes in bone marrow tissue samples. **H** Examples of SLFN12-negative and (**I**) SLFN12-positive and (**J**) weak, **K **intermediate, and (**L**) strong CD117 staining in GISTs. Each field of view corresponds to 0.2 mm × 0.2 mm

Immunohistochemical staining of PDE3A was first investigated in healthy colon tissue. We observed strong PDE3A staining in interstitial cells of Cajal, the suggested precursors of GIST, and in mast cells (Fig. 1). Additionally, endocrine cells within villi, endothelial cells, and ganglia showed weaker expression. Contrary to a previous study [22], we did not observe staining in thrombocytes or megakaryocytes in two bone marrow samples, although histiocytes showed granular staining pattern.

An IHC analysis was conducted on the GIST series from Cambridge, where all 26 GISTs were positive for PDE3A staining. Of these, four (15.4%) had weak, 12 (46.2%) intermediate, and 10 (38.5%) strong staining (Supplementary Table 1). Similarly, in the Helsinki Biobank series, all GISTs (*n* = 147) expressed PDE3A with staining intensity varying; seven cases (4.8%) showed weak, 50 (34%) intermediate, and 90 (61.2%) strong staining. Weak to strong PDE3A expression was observed in all investigated GISTs (Fig. 1; Supplementary Fig. 1). Inter-rater agreement among the three pathologists for the results from the Helsinki Biobank series was assessed using Fleiss'kappa. The analysis yielded a kappa (κ) value of 0.692 (standard error = 0.048, 95% confidence interval = 0.60 to 0.79, *p* < 0.001), indicating substantial agreement between the raters.

### PDE3A mRNA expression in GIST and liposarcoma samples

**Fig. 2:**
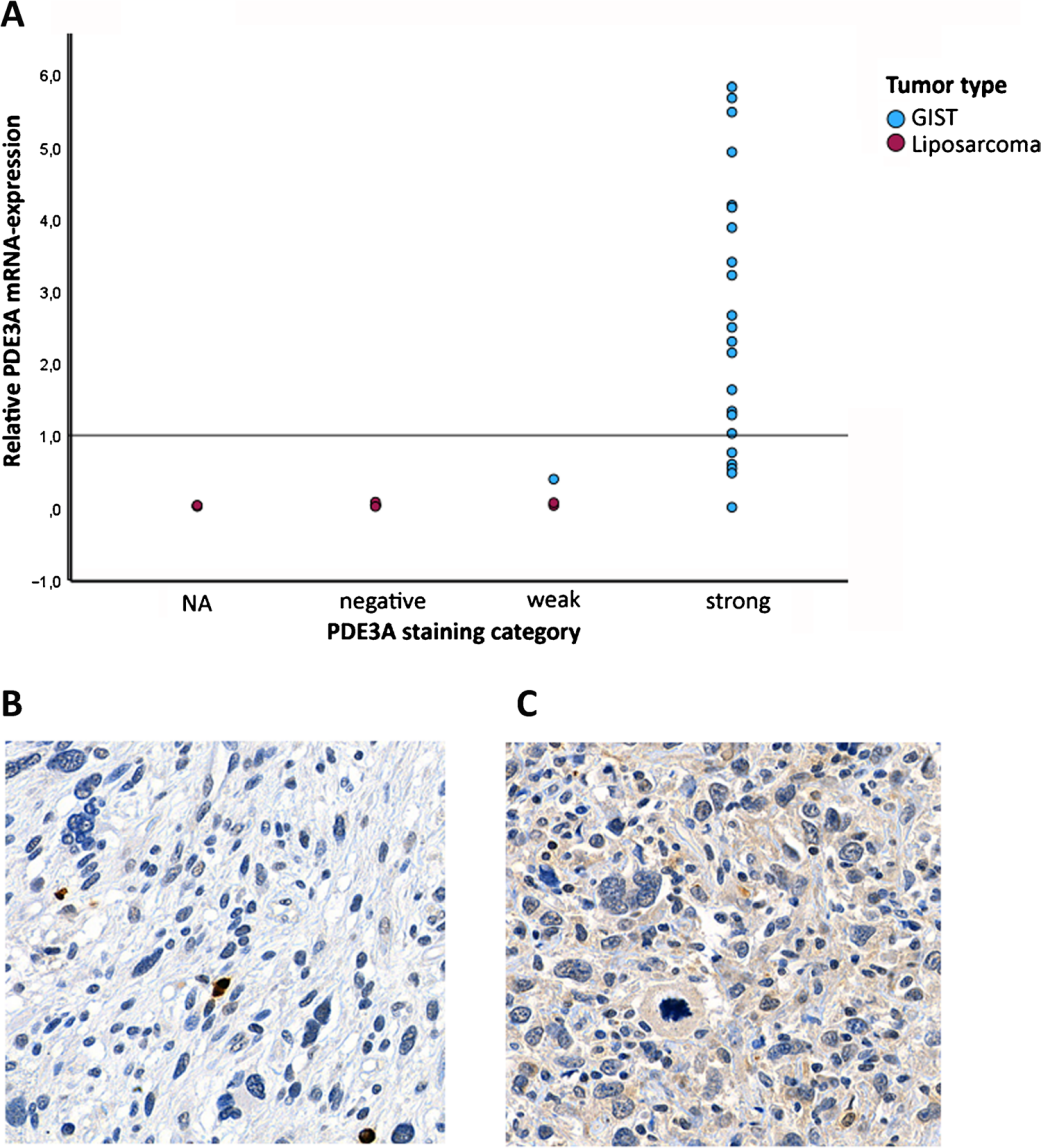
Comparison of relative PDE3A mRNA expression and PDE3A protein expression in 23 GIST and 10 liposarcoma tissues. Immunohistochemistry results were missing for three liposarcomas. Three GISTs with intermediate staining were grouped in the strong staining category. **A** PDE3A mRNA expression was significantly higher in GISTs than in liposarcomas (*p* < 0.001). Examples of (**B**) PDE3A-negative and (**C**) PDE3A weak staining in liposarcomas. Each field of view corresponds to 0.2 mm × 0.2 mm

Next, we investigated the correlation between PDE3A protein and mRNA expression in GIST and pleomorphic liposarcoma tissues. Pleomorphic liposarcomas were selected for the comparison as they contain frequently low PDE3A mRNA and protein expression [12]. RT-qPCR was successfully performed on 23 GIST and 10 liposarcoma samples. Tissue for IHC was available in all GISTs and in 7 liposarcomas. Relative mRNA expression levels were compared with RNA extracted from a FFPE GIST882 cell line. The results demonstrated that PDE3A mRNA expression is significantly higher in GISTs than in liposarcomas (median relative gene expression: 2.30 vs. 0.03, *p* < 0.001; Fig. 2; Supplementary Table 2). In addition, PDE3A protein and mRNA expression levels showed a strong positive correlation (*p* < 0.001).

### Association between PDE3A expression and clinicopathological factors and survival

Finally, we investigated the association between PDE3A expression and clinicopathological factors and survival in the Helsinki Biobank series (Table 1). Tumors with low PDE3A expression had a 4 to 5 times lower mitotic count than those with intermediate or strong staining (*p* = 0.007). Low PDE3A expression was also more frequently associated with metastasis at diagnosis than in intermediate and strongly staining tumors (28.6% vs. 8.0% vs. 3.3%, respectively; *p* = 0.04). In addition, PDE3A positive expression showed a strong correlation with CD117 (*p* < 0.001), but no significant association was present with sex, age at diagnosis, tumor location, mutation profile, or risk stratification category. Of cases, 65% co-expressed PDE3A and SLFN12 without a significant association (*p* = 0.083; Fig. 1).

**Fig. 3:**
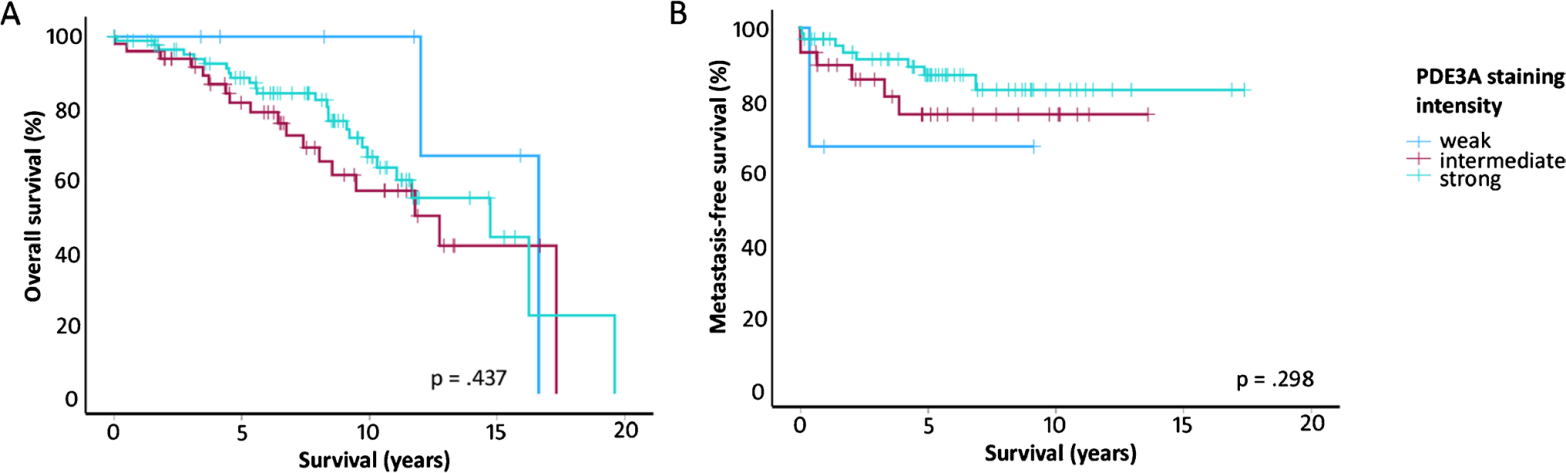
Association of PDE3A staining intensity with (**A**) overall survival and (**B**) metastasis-free survival in GISTs

Median follow-up time in the series was 7.5 years (range 1 day to 19.6 years) for overall survival and 5.1 years (range 24 days to 17.5 years) for metastasis-free survival. PDE3A expression was not associated with overall survival (*p* = 0.437) or metastasis-free survival (*p* = 0.298; Fig. 3).

## Discussion

Many studies indicate that PDE3A is a promising target for personalized GIST therapy, but its role as a diagnostic or prognostic biomarker has not yet been well established. Here, we investigated PDE3A expression levels and immunohistochemical staining patterns using a novel PDE3A-specific antibody in 173 GIST samples. We observed homogeneous PDE3A staining in all GISTs (100%) with intermediate or high expression detected in 94%. In addition, low expression was associated with a lower mitotic count in tumor cells and an increased frequency of metastases at diagnosis but not with metastasis-free or overall survival.

PDE3A is phosphodiesterase that regulates cellular levels of cyclic adenosine phosphate (cAMP). It is expressed in several healthy tissues, including cardiac myocytes, vascular smooth muscles, platelets, and ICCs, where it contributes to heart contractility, vascular tone, platelet aggregation, and ICC network development [13, 23]. In our study, we observed high and frequent PDE3A expression, consistent with previous findings in GIST [10–12, 17]. PDE3A mRNA levels were also elevated and correlated with IHC staining intensity. Strong staining was also detected in ICCs within colon tissue, while most surrounding tissue types, excluding neuroendocrine cells of colon villi, mast cells, and ganglia cells, showed little or no staining.

A previous study reported widespread PDE3A staining in non-neoplastic colon epithelium and underlying connective tissue, but the authors suggested that this was likely non-specific staining [24]. However, PDE3A positivity in ganglia and neuroendocrine cells has been confirmed in other studies. PDE3A mRNA expression has been detected earlier in superior cervical ganglia in rats [25], and protein expression is reported to be widespread in cells of the epithelial layer and sebaceous glands of labia, including interstitial or neuroendocrine cells [25, 26]. Interestingly, we did not detect previously reported PDE3A staining in platelets or megakaryocytes [22]. This discrepancy may result from differences in antibody clone specificity or detection thresholds for protein expression in IHC. A comparative analysis using different antibodies, as well as cell-specific detection of PDE3A using mRNA in situ hybridization or single-cell RNA sequencing, may be necessary to clarify these differences.

Since the primary anti-tumor effect of PDE3A modulators depends on drug-induced PDE3A–SLFN12 complex formation, assessing co-expression of both proteins is considered important for patient stratification. To address this, we assessed also SLFN12 expression using IHC and found co-expression in 65% of GISTs, which is notably lower than the 92% co-expression reported earlier [13]. This discrepancy may also stem from differences in antibody clones and IHC protocols between studies.

Recent reports suggest that PDE3A expression alone may be a key determinant of therapeutic response, with PDE3A modulator efficacy observed even in tumors with SLFN12 expression below the detection limit of IHC or immunoblotting [11, 21, 27]. One possible reason for the low SLFN12 detection rate is the inherently low basal expression of SLFN12 and its constant degradation in cells. Therefore, mRNA in situ hybridization has been proposed as a potentially more reliable method for estimating SLFN12 expression in tumor tissue [17].

Although most GISTs appear to co-express PDE3A and SLFN12, and preclinical models have shown that these tumors are sensitive to PDE3A-targeting treatment, further research is needed to define PDE3A and SLFN12 expression thresholds that may predict drug response. Additionally, the potential role of other biomarkers guiding therapy decisions, such as co-chaperon protein aryl hydrocarbon receptor-interacting protein (AIP) required for PDE3A-SLFN12 complex formation by DNMDP, remains unclear [28]. While patient stratification might not be necessary in GISTs due to a high co-expression rate, exploring diagnostics approaches could be beneficial for other tumor types, such as soft-tissue sarcomas, melanoma, and ovarian carcinoma, where PDE3A expression is less frequent [10–12].

Our study has some limitations. Although we observed a statistically significant association between PDE3A expression and both the number of mitoses and the presence of metastatic disease, these results should be interpreted with caution. Only seven GISTs in our series showed weak PDE3A positivity. Therefore, the findings should be validated in a larger, independent GIST cohort. An additional large sample cohort would also help assess whether staining differences could arise due to variations in sample fixation across different pathology laboratories.

In conclusion, PDE3A is uniformly expressed in the majority of GIST samples and that its protein expression can be reliably assessed using the monoclonal mouse antibody clone 230-F8-G1 and immunohistochemistry in FFPE tissue samples. Future studies are warranted to define expression thresholds and to determine the frequency of PDE3A expression across various cancer types, which may help to identify patients most likely to benefit from PDE3A-targeting therapies should these modulators enter clinical use.

## Supplementary Information

Below is the link to the electronic supplementary material.

Supplementary file1 (DOCX 14 KB)

Supplementary file2 (DOCX 19 KB)

ESM 3 Overview of PDE3A staining in a GIST tissue microarray (PNG 1.10 MB)

High Resolution Image (TIF 593 KB)

## Data Availability

Tissue samples, clinical data and the results were obtained from Helsinki Biobank as part of the study and are available upon reasonable request through Helsinki Biobank (project number HBP20210145). For more information, please visit [https://www.helsinginbiopankki.fi/]. All relevant data considering data from the GIST series of National Paediatric and Adult Wild-Type GIST and GIST Clinic at Cambridge University Hospitals NHS Foundation Trust, UK, are included in the article and supplemental information.
