# Supplementary material for: Phosphodiesterase 3 A expression in gastrointestinal stromal tumors
Source: Virchows Arch. 2025 Jun 18;487(5):983–91. doi: 10.1007/s00428-025-04150-1 (PMC12647229; doi:10.1007/s00428-025-04150-1)
Supplement: Supplementary file 1 — Supplementary file1 (DOCX 14 KB) [file 428_2025_4150_MOESM1_ESM.docx]

Supplementary Table 1. PDE3A staining in 26 GISTs.

|  | **PDE3A staining intensity** | | |
| --- | --- | --- | --- |
| **Mutated gene** | **Weak (n)** | **Intermediate (n)** | **Strong (n)** |
| *SDHx* | 1 | 6 | 5 |
| *NF1* | 1 | 1 | 2 |
| *KIT* | 0 | 4 | 2 |
| *PDGFRA* | 2 | 1 | 1 |
