# Supplementary material for: Phosphodiesterase 3 A expression in gastrointestinal stromal tumors
Source: Virchows Arch. 2025 Jun 18;487(5):983–91. doi: 10.1007/s00428-025-04150-1 (PMC12647229; doi:10.1007/s00428-025-04150-1)
Supplement: Supplementary file 2 — Supplementary file2 (DOCX 19 KB) [file 428_2025_4150_MOESM2_ESM.docx]

Supplementary Table 2. PDE3A immunohistochemistry and qPCR results for GIST and liposarcoma samples.

| ID | Mutation | Tumor | PDE3A intensity | Relative PDE3A mRNA expression | | | |
| --- | --- | --- | --- | --- | --- | --- | --- |
| G029 | KIT11 | GIST | 3 | 0,0083 |  |  |  |
| G033 | KIT11 | GIST | 2 | 1,02555 |  |  |  |
| G036 | KIT11 | GIST | 3 | 3,2179 |  |  |  |
| G038 | wt | GIST | 1 | 0,39103 |  |  |  |
| G039 | KIT11 | GIST | 3 | 1,32987 |  |  |  |
| G040 | KIT11 | GIST | 2 | 0,59864 |  |  |  |
| G043 | KIT11 | GIST | 3 | 5,47847 |  |  |  |
| G044 | PDGFRA18 | GIST | 3 | 0,53984 |  |  |  |
| G045 | KIT11 | GIST | 3 | 5,67359 |  |  |  |
| G047 | KIT11 | GIST | 3 | 0,47509 |  |  |  |
| G049 | wt | GIST | 3 | 4,1862 |  |  |  |
| G050 | wt | GIST | 3 | 3,87423 |  |  |  |
| G052 | PDGFRA18 | GIST | 3 | 0,75951 |  |  |  |
| G053 | PDGFRA18 | GIST | 3 | 2,14171 |  |  |  |
| G054 | KIT11 | GIST | 3 | 4,15386 |  |  |  |
| G057 | KIT9 | GIST | 3 | 2,29635 |  |  |  |
| G059 | KIT11 | GIST | 2 | 2,49369 |  |  |  |
| G060 | PDGFRA18 | GIST | 3 | 1,62793 |  |  |  |
| G061 | KIT11 | GIST | 3 | 3,4003 |  |  |  |
| G062 | KIT11 | GIST | 3 | 4,92349 |  |  |  |
| G063 | KIT11 | GIST | 3 | 2,65991 |  |  |  |
| G064 | wt | GIST | 3 | 5,82561 |  |  |  |
| G065 | PDGFRA18 | GIST | 3 | 1,28105 |  |  |  |
| L112 |  | LPS | not available | 0,01375 |  |  |  |
| L117 |  | LPS | 0 | 0,03453 |  |  |  |
| L180 |  | LPS | 0 | 0,03222 |  |  |  |
| L19 |  | LPS | not available | 0,01601 |  |  |  |
| L207 |  | LPS | not available | 0,02791 |  |  |  |
| L23 |  | LPS | 1 | 0,02418 |  |  |  |
| L4 |  | LPS | 0 | 0,02438 |  |  |  |
| L46 |  | LPS | 0 | 0,07222 |  |  |  |
| L72 |  | LPS | 0 | 0,01101 |  |  |  |
| L75 |  | LPS | 1 | 0,06281 |  |  |  |
