# Supplementary figures and images for: Phosphodiesterase 3 A expression in gastrointestinal stromal tumors
Source: Virchows Arch. 2025 Jun 18;487(5):983–91. doi: 10.1007/s00428-025-04150-1 (PMC12647229; doi:10.1007/s00428-025-04150-1)

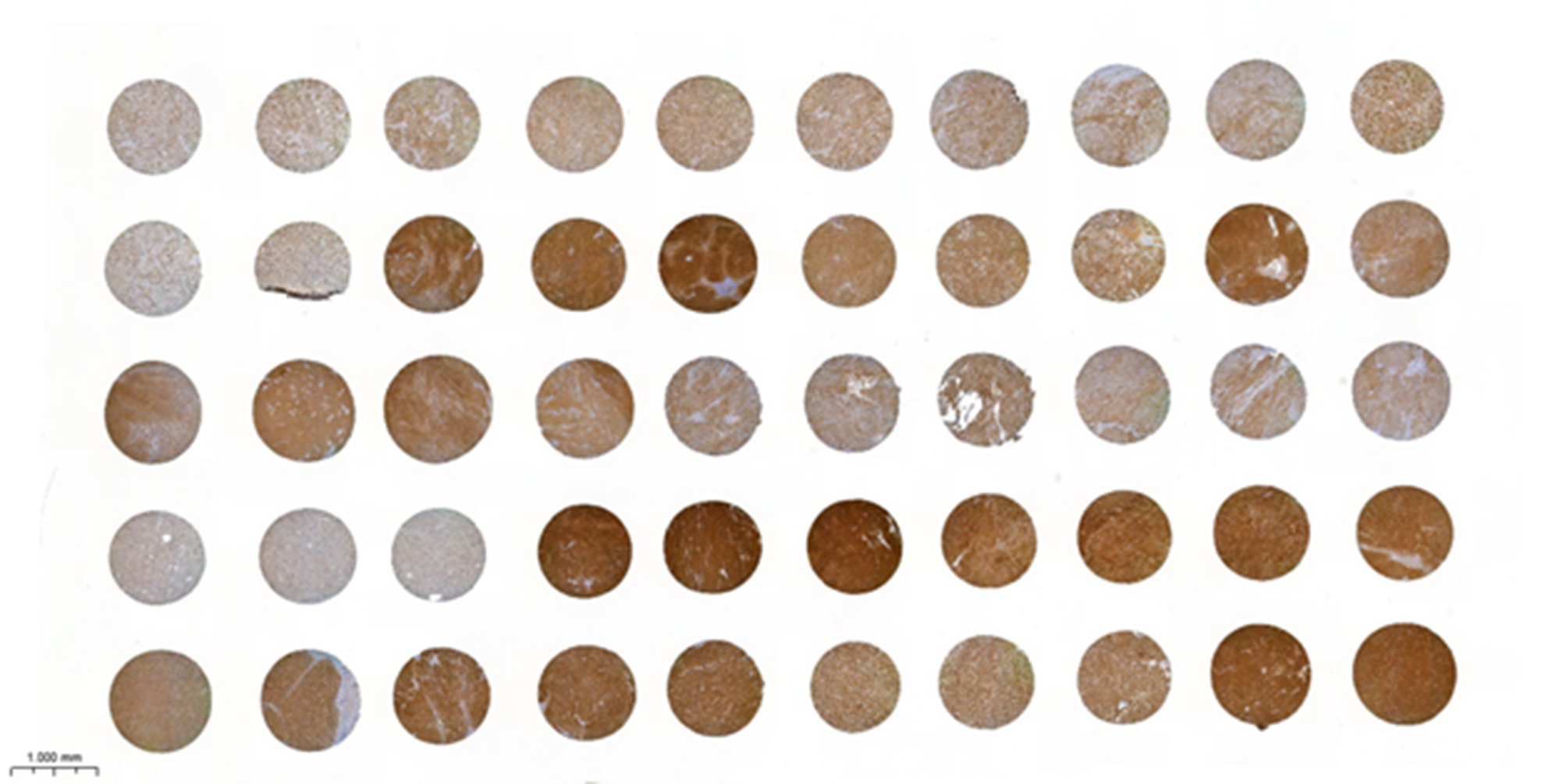

Supplement: Supplementary file 3 — Overview of PDE3A staining in a GIST tissue microarray (PNG 1.10 MB) [file 428_2025_4150_Fig4_ESM.png]

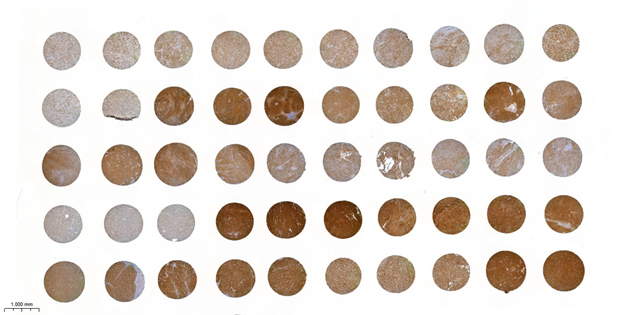

Supplement: Supplementary file 4 — High Resolution Image (TIF 593 KB) [file 428_2025_4150_MOESM3_ESM.tif]
